# Global coastal wind hazard maps from the CHAZ tropical cyclone model

**DOI:** 10.1038/s41597-025-06452-0

**Published:** 2026-01-17

**Authors:** Simona Meiler, Chia-Ying Lee, Suzana J. Camargo, Adam H. Sobel

**Affiliations:** 1 Civil and Environmental Engineering, Stanford University, Stanford, CA USA; 2 Institute for Environmental Decisions, ETH Zurich, Zurich, Switzerland; 3 Lamont-Doherty Earth Observatory, Columbia University, Palisades, NY USA; 4 Columbia Climate School, Columbia University, New York, NY USA; 5 Department of Applied Physics and Applied Mathematics, Columbia University, New York, NY USA

**Keywords:** Climate-change impacts, Natural hazards

## Abstract

Synthetic tropical cyclone (TC) models have become essential tools for assessing TC hazard and risk, due to both the shortness of observational records and, now, the influence of climate change. However, the direct output from these models, TC track sets, requires additional processing to become useful for impact assessments beyond traditional climate science and meteorological research. Converting TC tracks into hazard maps condenses complex information on TC intensity and frequency into widely accessible formats, facilitating diverse impact analyses. Here, we present global coastal wind risk maps generated using the Columbia TC hazard model (CHAZ) for both present-day and future climate conditions. Climate scenario-driven data are provided for a recent historical period (1995–2014) and two future periods, mid-century (2041–2060) and end-of-century (2081–2100); a reference data set downscaled from an observation-based reanalysis for 1981–2019 is also provided. These hazard maps depict TC intensity exceedance probabilities and return periods, providing valuable insights to policymakers, planners, and researchers focused on disaster preparedness.

## Background & Summary

Tropical cyclones (TCs) are among the most damaging weather-related hazards worldwide, with far-reaching consequences for people, infrastructure, and economies, particularly in coastal regions. Despite their importance, observational records of TCs are too short to reliably estimate the frequency and intensity of rare, high-impact events, and they are limited in their ability to inform long-term risk assessments under future climate conditions^1^. As the demand grows for climate-informed hazard data to support adaptation planning and disaster risk reduction, synthetic TC event sets offer a valuable solution by simulating thousands of plausible events across varying climate scenarios.

Several recent efforts have advanced synthetic TC hazard modeling. One of these is the Columbia HAZard model (CHAZ), a statistical-dynamical model that simulates large sets of synthetic TC events^2^, and whose code is open-source. Each event is a synthetic TC, defined by a “track” which gives its intensity and other features at a set of spatial points on the earth’s surface, denoting the position of the storm’s center at evenly spaced points in time from the storm’s formation to its dissipation. By including many more storms than are present in observations, probabilities of rare events that have not occurred — but, according to the model, could occur — can be estimated from such large synthetic track sets. Further, if the model is appropriately informed by climate simulations or projections, the influence of human-induced climate change on TC hazard and risk can be assessed.

Synthetic TC track sets as described above have been used in various applications, including probabilistic risk assessments. However, working directly with such track sets typically requires specialized expertise and computational resources to process and analyze large volumes of track-level data. This can limit their accessibility and application in other fields, such as human mobility or economic modeling, where TC risk may play an important role but where users may lack the tools or background to work directly with track-level data. To address this gap, we provide spatially explicit hazard maps of local exceedance intensities and return periods derived from CHAZ track sets. These ready-to-use hazard layers aim to facilitate broader interdisciplinary use beyond traditional climate risk modeling. Similar in spirit to the Synthetic tropical cyclOne geneRation Model (STORM) dataset by Bloemendaal *et al*.^3^, which offers a globally consistent set of wind return periods under present-day and future climate conditions, our dataset serves as the CHAZ-based counterpart.

We leverage the open-source climate risk modeling platform CLIMADA (CLIMate ADAptation)^4^ to generate gridded wind fields from the CHAZ synthetic tracks, which were downscaled from a range of global climate models (GCMs), emission scenarios, and for different time periods. Based on these TC wind fields, we compute hazard maps of local exceedance intensity and return periods. The resulting dataset comprises both a historical baseline derived from the European Centre for Medium-Range Weather Forecasting (ECMWF)’s fifth-generation climate reanalysis dataset (ERA5)^5^ and future projections based on Coupled Model Intercomparison Project Phase 6 (CMIP6) models^6^, processed through a consistent and reproducible workflow. As the wind fields are calculated with land-focused risk assessments in mind, the underlying horizontal resolution is finer over land (300 arcseconds; ca. 9.3 km at the equator) than over the ocean (1 degree). Accordingly, the dataset is best understood as representing coastal TC wind hazard.

A first version of the dataset has already been used in multiple applied projects. First, it serves as the TC component of the Natural Hazards Climate Change Projections platform, an interactive web tool developed by the Columbia University’s National Center for Disaster Preparedness (NCDP) that presents future hazard projections, including for wildfires, tornadoes, and sea-level rise, across the United States^7^. Second, it powers the Climate Migration Dashboard, an interactive portal for exploring comprehensive data on climate change–driven migration and its socio-economic impacts worldwide^8^. Third, the dataset supports the Greater Caribbean Climate Mobility Initiative (GCCMI), specifically modeling future climate-related mobility and displacement scenarios across the Caribbean^9^. Fourth, it provides inputs to model how education can mitigate climate-related poverty for a forthcoming World Bank Development Group working paper^10^.

Together, these applications highlight the dataset’s value for long-term climate risk assessment, adaptation planning, and regional policy design. By providing spatially explicit and climate-informed TC hazard metrics in standardized formats, the dataset facilitates integration into broader risk assessments, impact modeling, and decision-making workflows. All data are made openly available, with accompanying documentation to support reuse across disciplines and sectors.

## Methods

### Columbia HAZard model

The Columbia HAZard model (CHAZ) is a statistical-dynamical TC model designed for computational efficiency, capturing key physical relationships between TCs and their large-scale environmental conditions^2^. Synthetic TCs are initialized through random seeding, based on the Tropical Cyclone Genesis Index (TCGI) developed by Camargo *et al*.^11^ and Tippett *et al*.^12^, with the formulation fitted to ERA5 reanalysis. For future projections, the TCGI derived from GCM output is bias-corrected using ERA5 climatology prior to downscaling^13,14^. These steps differ from earlier CHAZ applications, where the genesis model was fitted to ERA-Interim and no bias correction was applied^15^. Following genesis, storm trajectories are simulated using a beta-and-advection model^16^ driven by monthly mean environmental wind fields, along with a statistical parameterization of sub-monthly variability. TC intensity is modeled along each track using an autoregressive linear model that accounts for environmental predictors such as potential intensity, vertical wind shear, and mid-level relative humidity, as well as a stochastic term to capture intrinsic variability^17^. The CHAZ intensity component has been extensively validated in previous studies^15,17,18^. It captures the mean intensity response to large-scale environmental predictors, while unresolved nonlinear processes are represented statistically through a stochastic perturbation derived from model residuals, allowing the model to reproduce observed distributions of lifetime maximum and landfall intensities across basins.

We use two sets of CHAZ simulations to construct the TC hazard maps. The first set, used in Meiler *et al*.^1^, is based on 39 years (1981–2019) of ERA5 reanalysis data^5^. CHAZ does not use ERA5 wind fields directly. Instead, it relies on monthly mean ERA5 variables, along with statistics of sub-monthly eddy wind covariances that drive the beta-and-advection model, to represent large-scale environmental conditions. These fields serve only as inputs to the statistical–dynamical modeling framework that generates synthetic TCs, thereby avoiding biases associated with the coarse resolution of ERA5 wind speeds near the storm core. CHAZ is downscaled with 10 independent realizations of the TC genesis and track model. For each realization, 40 ensemble members are generated using the intensity model, resulting in a total of 400 ensemble members for the historical period.

The second set of simulations, as used in Meiler *et al*.^19^, employs CHAZ^2,13,15^ to generate synthetic TC event sets for three future scenarios from the Shared Socioeconomic Pathways (SSPs): SSP2-4.5, SSP3-7.0, and SSP5-8.5, representing low, medium, and high greenhouse gas emissions, respectively. As with ERA5, CHAZ does not use raw GCM wind fields directly but instead relies on monthly mean large-scale variables and their sub-monthly covariance statistics to drive the beta-and-advection and intensity models. These simulations are based on outputs from six CMIP6 GCMs (CESM2^20^, CNRM-CM6-1^21^, EC-Earth3^22^, IPSL-CM6A-LR^23^, MIROC6^24^, and UKESM1-0-LL^25^). We note that additional CHAZ track sets based on other GCMs are available^13^.

All GCM-driven simulations also incorporate two alternative formulations of the moisture variable in the TCGI component of CHAZ^15^. Both variables represent atmospheric humidity but differ in formulation: the column-integrated relative humidity (CRH) expresses the ratio between actual and saturated water vapor, while the saturation deficit (SD) represents their absolute difference^11,15,26^. In the current climate, both behave similarly, but under warming, CRH tends to remain nearly constant as SD decreases with increasing saturation vapor pressure. This difference leads to diverging responses of the TCGI in future climates, typically increasing when based on CRH and decreasing when based on SD. As there is no theoretical or empirical consensus on the direction of global TC frequency change, CHAZ includes both formulations to bracket this uncertainty. While a humidity variable is essential in any TC genesis model, CHAZ is distinct in explicitly incorporating two alternative formulations to represent this uncertainty in TC genesis under climate change.

For each combination of GCM, emission scenario, and TCGI variant, CHAZ is run with 10 genesis realizations. Each genesis realization is paired with 40 ensemble members of the intensity model. To balance computational efficiency with statistical robustness for TC risk analysis, we retain all 10 genesis realizations but randomly sample 8 out of the 40 intensity ensembles, resulting in 80 ensemble members per configuration. We note that variability across genesis realizations contributes more strongly to the overall ensemble spread than variability among intensity members; the resulting 80-member ensemble therefore captures the total spread effectively. We use 20-year time windows, 1995–2014, 2041–2060, and 2081–2100, to represent TC event sets for the present-day and two future time periods. The ERA5 and GCM baselines serve different purposes: ERA5 (1981–2019) provides a longer historical reference for present-day analyses, whereas the GCM baseline (1995–2014) provides a consistent 20-year reference period against which future changes are evaluated. The two baselines are therefore not used interchangeably but each in the context for which it was designed.

A complete overview of available climate scenario-driven CHAZ simulations, including the combinations of GCMs, scenarios, TCGI variants, and time periods, is provided in Table 1 in the Data Records section.

**Table 1:** Summary of available GCMs, scenarios, time periods, and TCGI inputs.

Climate model	Emission scenario	Period	TCGI variable
CESM2^20^	SSP2-4.5	base (1995–2014)	CRH
CNRM-CM6-1^21^	SSP3-7.0	fut1 (2041–2060)	SD
EC-Earth3^22^	SSP5-8.5	fut2 (2081–2100)	
IPSL-CM6A-LR^23^			
MIROC6^24^			
UKESM1-0-LL^25^			

### CLIMADA and wind field computation

We use the open-source, probabilistic climate risk modeling platform CLIMADA^4^ to translate the CHAZ TC tracks into global wind hazard layers. Developed as a community project, CLIMADA is written in Python (version 3.11 + for this study) and is publicly available under the GNU General Public License v3. In this work, we use CLIMADA v6.0.2-dev.

In CLIMADA, the TC hazard is represented as a two-dimensional gridded wind field derived by combining synthetic TC track sets with a parametric wind model. The wind model calculates 1-minute sustained winds at 10 meters above ground as the sum of a circular wind component, based on the Holland (2008) parameterization^27^, and a translational wind component arising from the forward motion of the storm. To account for the spatial decay of the translational wind speed with distance from the cyclone center, we apply an attenuation factor following Geiger *et al*.^28^. Since the CHAZ track data do not provide central pressure or radius of maximum winds, these parameters are statistically inferred from available variables using relationships derived from the IBTrACS observational dataset, based on ordinary least squares regression fits to latitude, longitude, and maximum wind speed data from the 1980–2019 period.

Wind fields are computed globally at 300 arcsecond resolution over land and 3600 arcseconds over the ocean. CLIMADA stores and uses the lifetime maximum wind speed at each grid cell as the hazard metric. Values below 34 knots (17.5 m/s), based on the 1-minute sustained wind speed at 10 m height, are excluded from further analysis, consistent with standard TC hazard thresholds.

### Frequency bias correction

The CHAZ TC event sets require a frequency bias correction to ensure realistic annual TC occurrence rates^13^. We applied this correction separately to each synthetic event set generated by each GCM, prior to calculating hazard return periods and exceedance frequency maps. For each TC basin, we obtained the average annual frequency for the period 1980–2018 from the IBTrACS record, as reported in Bloemendaal *et al*. (2020, Table 3)^29^. The uncorrected TC frequency in CHAZ is given by dividing the number of synthetic TCs, *N*, by the total number of simulated years, *Y*, where $Y=\text{ensemble members}\times \text{years per member}$:

$${f}_{\mathrm{sim}}=\frac{N}{Y}$$

A correction factor $c$ is then derived as the ratio of the observed frequency ${f}_{\mathrm{obs}}$ to the simulated frequency:

$$c=\frac{{f}_{\mathrm{obs}}}{{f}_{\mathrm{sim}}}$$

This factor is applied uniformly to all synthetic TCs by assigning each storm an annual frequency weight $w$:

$$w=\frac{c}{Y}=\frac{{f}_{\mathrm{obs}}}{N}$$

The weights are then held constant in all climate scenarios and time periods. Holding the per-storm weight constant ensures comparability across time periods, while allowing total basin frequency to vary naturally with the number of simulated storms in each climate scenario. In other words, any projected change in overall TC frequency emerges from differences in storm counts generated by CHAZ across scenarios, not from changes in the fixed weight itself. This approach retains the original distribution of storm intensities and tracks, while aligning the overall storm occurrence rate with observed climatology. It is applied at the basin level and does not explicitly account for spatial, seasonal, or intensity-specific variations, which is appropriate for analyses treating each basin’s hazard as representative of its overall climatology rather than sub-seasonal variability. Potential biases in the intensity distribution are already mitigated within the CHAZ model, which realistically reproduces the full spectrum of storm intensities and corrects for the underrepresentation of very intense events typical of GCM-based simulations^2,15^. It is nonetheless a rather limited form of bias correction, in that it addresses only basin-wide frequency, rather than (for example) hazard or risk along any section of coastline. Additional bias correction might well be desirable for some applications.

### Local exceedance intensity maps

To derive spatially explicit estimates of wind intensity associated with different return periods, we use the local_exceedance_intensity method in CLIMADA’s TC hazard module. This method computes, for each grid cell, the wind speed associated with an average exceedance frequency of once every $T$ years—that is, the intensity with a 1/*T* annual probability of being exceeded— yielding local exceedance intensity maps. To determine these values, the method first examines the distribution of wind intensities at each grid cell (centroid) across all synthetic TCs in the event set. It ranks the events by their intensity at that location and calculates the cumulative exceedance frequency for each intensity level by summing the frequencies of all events exceeding that value. The return period associated with a given intensity is then defined as the inverse of this cumulative frequency. Since intensities corresponding to specific return periods may not be directly observed in the event set, CLIMADA infers them through interpolation or extrapolation.

CLIMADA supports several interpolation and extrapolation approaches, each with different assumptions about extreme tail behavior and data sparsity. For instance, the interpolate method fits a smooth curve within the observed range of intensities, while extrapolation methods extend estimates beyond observed data with varying assumptions. In this study, we use the extrapolate_constant method, a conservative and robust choice, particularly suited for regions with few high-intensity events. Return periods beyond the maximum observed in the event set are assigned the highest observed intensity, while those below the smallest observed are assigned an intensity of zero. This conservative approach ensures that estimated intensities remain within the physically and statistically plausible range of the modeled event set, avoiding unrealistically high values in data-sparse regions. We compute local exceedance intensity maps for return periods of 10, 25, 50, 100, 250, and 1000 years.

### Local return period maps

To assess how frequently wind speeds exceed predefined intensity thresholds at each location, we use the local_return_period method in CLIMADA. This method calculates, for each grid cell, the return period of exceeding a given wind speed threshold by summing the frequencies of all events exceeding that threshold and taking the inverse. In this context, the return period represents the average time interval between exceedances, corresponding to a $1/T$ annual probability that the threshold will be exceeded in any given year, rather than implying that it occurs exactly once within a $T$-year period. Similar to exceedance intensity mapping, multiple methods are available to handle values outside the empirical range. Here too, we apply the extrapolate_constant. Thresholds exceeding the maximum observed intensity at a given grid cell are assigned an infinite return period, while thresholds below the smallest observed intensity are assigned a return period of zero. The same conservative extrapolation principle is applied here as for the exceedance intensity maps, ensuring consistent treatment of extreme tail behavior and stable results in regions with limited storm sampling. We compute local return period maps for two wind speed thresholds: 33 m s^−1^ (corresponding to Category 1) and 50 m s^−1^ (Category 3 and higher) storms on the Saffir–Simpson Hurricane Wind Scale^30^.

## Data Record

The CHAZ wind risk maps are available via Columbia University’s Dryad repository: 10.5061/dryad.qfttdz0vz^31^. The dataset contains global TC wind hazard maps derived from CHAZ simulations and processed as outlined in the Methods section. The dataset includes local exceedance intensity and return period maps for various GCMs, emission scenarios, moisture variables used in the TCGI, and time periods. All outputs are provided in three formats: point-based NetCDF and CSV files, and gridded NetCDF rasters.

The data repository is organized as follows. Files are grouped first by hazard metric (exceedance_intensity or return_periods), then by output format (csv, nc, or raster.nc). Within each format, files are separated by data origin:

- **ERA5**: A historical baseline dataset based on CHAZ simulations using ERA5 reanalysis data (1981–2019). Filenames use ERA5 as the model identifier.
- **per-GCM**: Hazard maps based on individual GCM simulations. Files are grouped first by GCM, and then by SSP. Each simulation contains 80 ensembles and is marked with the 80ens ensemble identifier.

In all folders, the moisture variable formulation used in the TCGI, either column-integral relative humidity CRH or saturation deficit SD, is embedded in the filename and not used for subfolder separation, allowing users to easily filter or compare the two variants within a scenario.

### Data format and naming convention

Each file is named according to the following convention:

TC_“basin”_“res”_CHAZ_“GCM”_“period”_“scenario”_“ens”_“TCGI”_“wind”_“map”.“format”

Where fixed components include:

- basin: always global
- res: spatial resolution, always 0300as (300 arcseconds ≈9.3 km at the equator)
- ens: number of ensembles (80ens)
- wind: wind model used, currently always H08 (Holland, 2008^27^)

Varying components include:

- map: either return_periods or exceedance_intensity
- format: one of csv, nc (point-based NetCDF), or raster.nc (gridded NetCDF)
- GCM, period, scenario, TCGI: representing the climate model, time window, emissions scenario, and TCGI moisture formulation (CRH or SD)

A summary of the GCMs, emission scenarios, time periods, and TCGI variants used is provided in Table 1. Local exceedance intensity and return period maps are available for all possible combinations.

## Technical Validation

We assess the robustness and illustrate the potential use of our CHAZ-derived coastal wind hazard maps through three complementary examples. These examples demonstrate how the dataset can be applied across a range of possible analyses to validate plausibility and illustrate its suitability for impact-oriented applications rather than to derive new scientific findings. Specifically, we perform: (1) a global check of 100-year exceedance intensities using CHAZ simulations from ERA5 reanalysis; (2) a regional comparison between ERA5-based and GCM-driven hazard maps — using the multi-model median of all six GCMs — under historical and future climates; and (3) a point-based comparison at six representative coastal cities spanning all major TC basins. Analyses (2) and (3) are carried out separately for exceedance intensity maps (Section Exceedance intensity maps) and return period maps (Section Return period maps), and for both CRH and SD formulations of the TCGI. Together, these examples illustrate that our workflow reproduces realistic spatial patterns, remains consistent across different data sources, and yields local estimates aligned with existing reanalysis products and published benchmarks, thereby confirming the dataset’s robustness and general applicability.

### Exceedance intensity maps

**Fig. 1:**
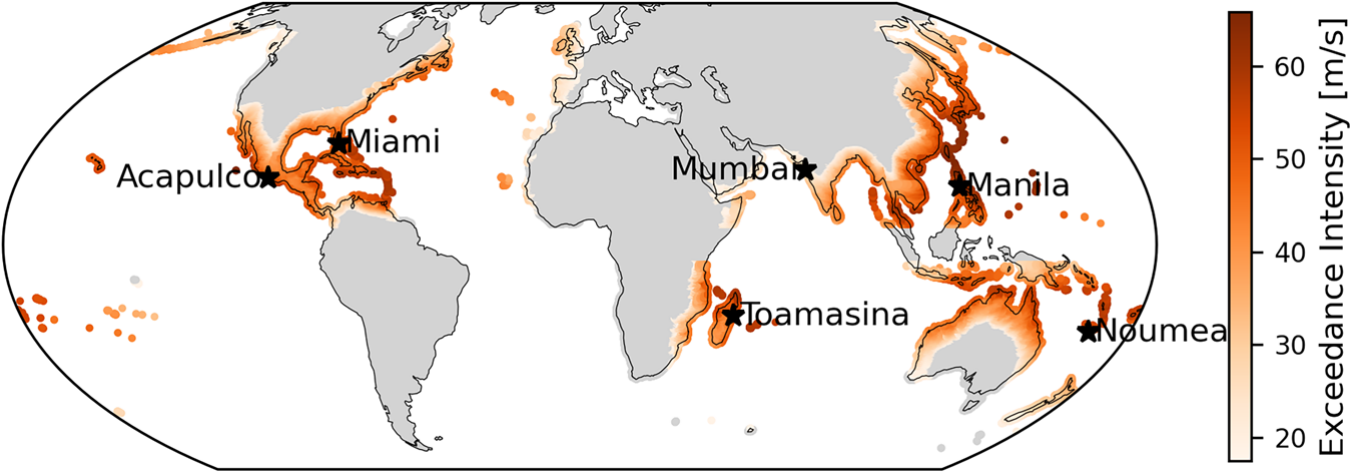
Global coastal 100-yr tropical cyclone wind exceedance intensities for the historical period. Spatially explicit estimate of wind intensities associated with a 100-yr return period from TC windfields downscaled from ERA5 reanalysis for 1981–2019. Local exceedance intensities over the ocean are not shown. The location of six major coastal cities in different basins is marked on the map. The respective exceedance intensity and return period values are listed in Tables 2, 3, Table S1, and Table S2.

We map global 100-year exceedance intensities from ERA5-downscaled wind fields in Fig. 1. Peak values exceed 60 m s^−1^ along well-known TC-prone coastlines in the western North Pacific, North Atlantic, and eastern North Pacific, with a decrease in intensity poleward and inland. This spatial gradient aligns with observational climatologies (e.g., the International Best Track Archive for Climate Stewardship, IBTrACS^32^) and previous hazard studies^3^, providing confidence in both the parametric wind field generation and the local exceedance intensity computation.

**Fig. 2:**
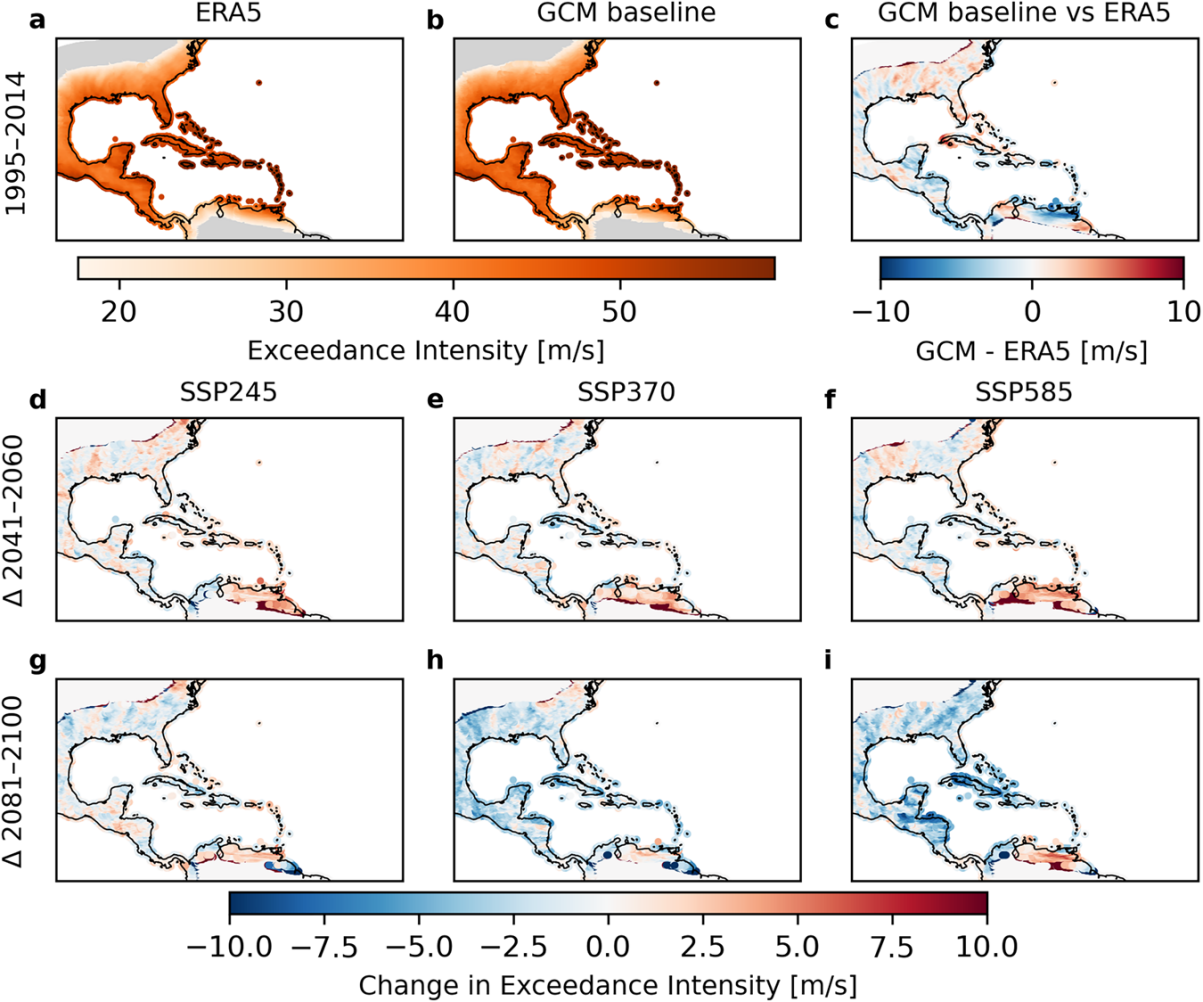
100-yr exceedance wind speeds (m/s) estimates for the Carribean - CRH TCGI. The top row shows exceedance intensity estimates for the present-day baseline from ERA5 reanalysis (**a**), the GCM baseline (**b**), and difference between ERA5 and GCM baselines (**c**). The middle (bottom) row shows difference plots for the middle (end) of the century relative to the present-day baseline for three climate scenarios (SSP2-4.5 (**d,g**), SSP3-7.0 (**e,h**), SSP5-8.5 (**f,i**)). We computed the multi-model median from the six GCMs, using the CRH TCGI.

To illustrate regional behaviour and assess internal consistency, we next focus on the Caribbean (Fig. 2 and Figure S1). The historical (1995–2014) exceedance intensities from the median GCM CHAZ baseline closely track those derived from ERA5, regardless of the TCGI moisture formulation. Differences between the median GCM baseline and ERA5 simulations remain within roughly ± 5 m s^−1^, confirming the reproducibility of spatial patterns across data sources.

Local exceedance intensity maps under various future climate projections are provided in the Fig. 2 and Figure S1d–i). These demonstrate that the dataset can be used to evaluate future TC hazard changes across emission scenarios, time periods and both TCGI formulations.

Finally, we evaluate 100-year exceedance intensities at six coastal cities: Acapulco, Manila, Miami, Mumbai, Nouméa, and Toamasina (Table 2 and Table S1), each representing a different TC basin. For both CRH and SD TCGI-based simulations, the ERA5-derived intensities lie within the multi-GCM baseline range in all but three instances (Manila under CRH; Mumbai and Toamasina under SD), and even those deviations are small relative to observational and intermodel uncertainty.

**Table 2:** 100-yr exceedance wind speeds (m/s) for six cities – CRH TCGI Listed are the modelled wind speeds corresponding to a 100-year return period in the historical period (ERA5 and GCM baseline) and two future windows (2041–2060 and 2081–2100) under three SSP scenarios (SSP2-4.5, SSP3-7.0, SSP5-8.5) using the CRH TCGI. Cities span six basins: Eastern Pacific (EP), North Atlantic (NA), North Indian Ocean (NI), South Indian Ocean (SI), South Pacific (SP), and Western Pacific (WP). The central value is the multi-model median; values in parentheses show the range (minimum to maximum) across individual GCMs.

	basin	ERA5 [m/s]	base [m/s]	ssp245 [m/s]	2041–2060 ssp370 [m/s]	ssp585 [m/s]	ssp245 [m/s]	2081–2100 ssp370 [m/s]	ssp585 [m/s]
**Acapulco**	EP	55.39	54.33 (51.03–57.71)	54.62 (52.36–58.19)	55.39 (44.36–57.03)	55.88 (49.28–57.47)	53.23 (50.65–57.61)	52.64 (50.36–60.79)	55.79 (48.19–61.29)
**Manila**	WP	51.59	54.22 (51.68–56.33)	53.54 (51.92–55.12)	54.27 (52.14–56.41)	55.53 (53.64–57.43)	52.77 (51.14–53.62)	52.77 (52.25–56.19)	55.02 (52.85–57.56)
**Miami**	NA	51.44	50.84 (48.23–54.62)	51.84 (48.24–55.01)	52.53 (49.26–59.05)	51.62 (46.69–52.73)	50.84 (47.44–53.82)	48.40 (46.34–53.84)	48.87 (43.57–57.92)
**Mumbai**	NI	27.83	31.06 (27.19–34.02)	32.34 (28.12–34.75)	30.20 (28.62–34.85)	33.41 (29.36–37.76)	32.90 (30.60–36.29)	32.15 (30.26–36.80)	33.10 (29.17–40.16)
**Noumea**	SP	52.96	53.28 (51.34–56.70)	55.39 (51.16–56.35)	55.27 (49.91–57.97)	56.16 (50.29–58.93)	54.91 (52.87–57.14)	54.85 (50.38–58.13)	54.23 (52.81–59.08)
**Toamasina**	SI	53.92	55.23 (51.24–59.10)	54.70 (0.00–56.32)	54.68 (0.00–57.19)	55.33 (0.00–57.44)	53.88 (0.00–56.48)	53.84 (4.28–56.07)	54.83 (21.76–59.55)

For future climate estimates, the late-century projections under SSP5-8.5 further confirm that the dataset behaves as expected from the underlying CHAZ simulations and published literature. CRH TCGI-based medians remain at or slightly above their baseline medians, whereas SD TCGI-derived medians fall below their baseline. CRH maxima often exceed the baseline median by several m s^−1^, while SD maxima stay near or below baseline, but SD minima drop well below, aligning with known TC frequency decreases for these simulations^13,15^. Additionally, the CRH TCGI-based estimates exhibit a larger intermodel range and thus higher model uncertainty than the SD counterparts.

Taken together, these results demonstrate that our approach produces consistent, basin-wide 100-year wind estimates across multiple scenarios and data sources, yielding median values that align with climatological expectations and well-bounded ranges that transparently bracket model uncertainty.

### Return period maps

We next evaluate our local return period maps for Category 1 (33 m s^−1^) wind intensities in a regional comparison in Southeast Asia and a point-based check at six coastal cities.

**Fig. 3:**
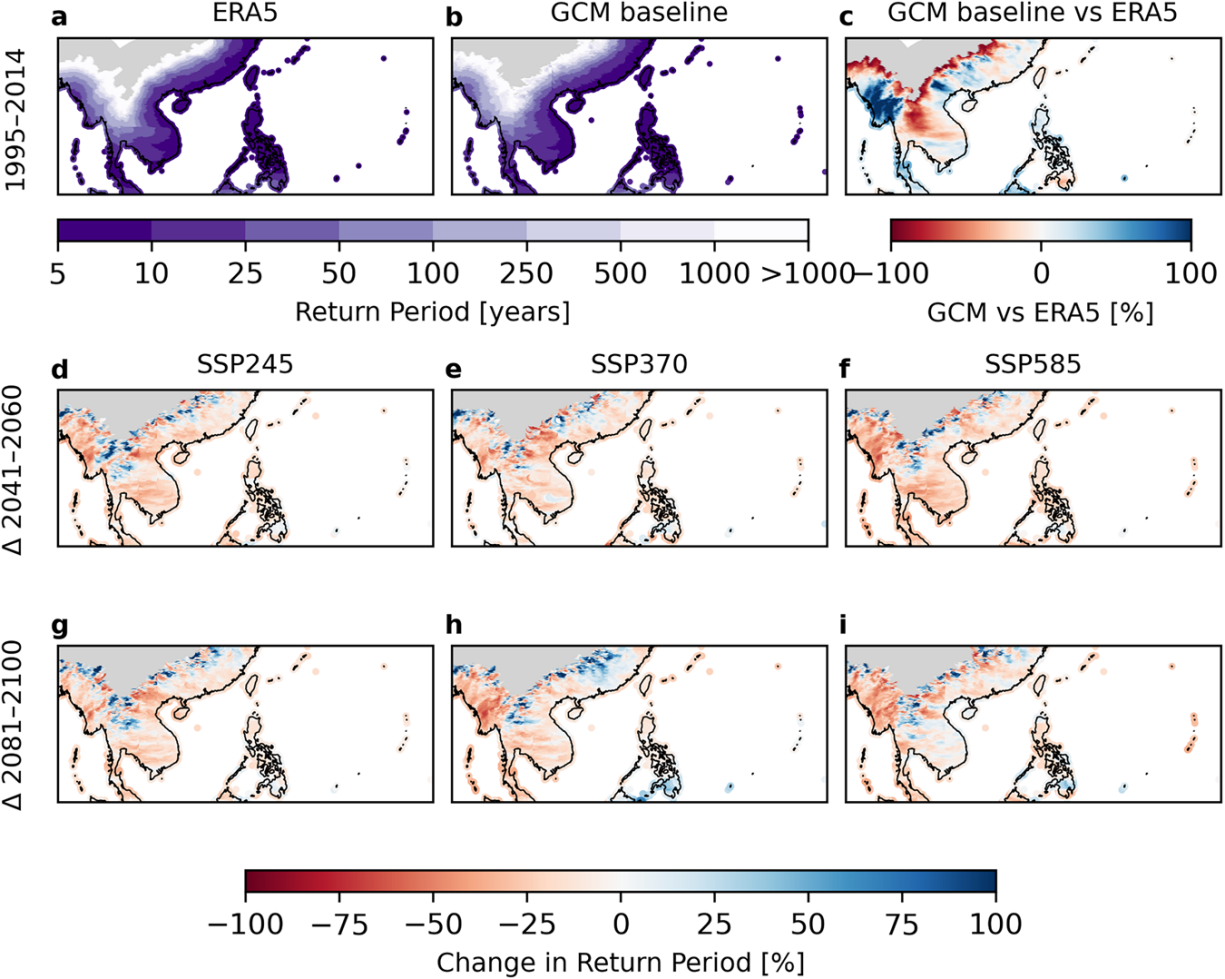
Return period (years) estimates for Cat. 1 TC wind intensities in Southeast Asia. The top row shows return period estimates for the present-day baseline from ERA5 reanalysis (**a**), the GCM baseline (**b**), and difference between ERA5 and GCM baselines (**c**). The middle (bottom) row shows difference plots for the middle (end) of the century relative to the present-day baseline for three climate scenarios (SSP2-4.5 (**d,g**), SSP3-7.0 (**e,h**), SSP5-8.5 (**f,i**)). We computed the multi-model median from the six GCMs, using the CRH TCGI.

We illustrate the use of the retun period maps in an example for Southeast Asia (Fig. 3 for CRH; Figure S2 for SD), the historical (1995–2014) return periods from the multi-model CHAZ baseline closely mirror those derived from ERA5, with median differences generally under $\pm$20%. The GCM median underestimates return periods (i.e., predicts more frequent Category 1 exceedances) along parts of the Philippine coast and western Thailand, while it overestimates them in Myanmar, peninsular Malaysia, southern Vietnam, and parts of the South China Sea. We also note isolated red patches appearing inland at the domain fringes (Fig. 3c; Figure S2c); these large relative differences reflect very few events in those areas, resulting in inherently less robust return period estimates and underscoring model limitations (cf. Usage Notes).

Mid-century (2041–2060) changes under all emission scenarios remain modest, typically within ${\rm{\pm }}$ 25%, whereas by 2081–2100 the shifts intensify, with most coastlines showing increased return periods (i.e., fewer storms meeting the threshold), particularly under SSP5-8.5 (Figure S2i). The SD variant again exhibits the largest increases, exceeding 100% in some areas, consistent with a decrease in cyclone frequency when the moisture variable in the TCGI is represented by SD^13,15^. These illustrations demonstrate that the dataset reproduces physically consistent changes across time horizons and emission scenarios and behaves in line with the underlying CHAZ simulations and published literature.

We compare Category 1 return periods at the same six cities: Acapulco, Manila, Miami, Mumbai, Nouméa, and Toamasina (Table 3 and Table S2). Under both TCGI formulations, ERA5-derived return periods agree within the ensemble range for three of six cities; ERA5-derived estimates in Nouméa and Toamasina fall just above the GCM maximum, while Mumbai Cat. 1 events have much higher return periods in the ERA5 simulations than GCM runs, reflecting sparse TC activity and elevated uncertainty in those basins. The picture for future simulations largely mirrors the findings described for the exceedance intensity estimates. We conclude that our return period maps accurately reproduce realistic spatial patterns in active basins, while also cautioning users about higher uncertainty in regions with infrequent storms.

**Table 3:** Return period (years) estimates for Cat. 1 TC wind intensities for six cities – CRH TCGI. Listed are the modelled return periods corresponding to a Cat. 1 TC wind intensity in the historical period (ERA5 and GCM baseline) and two future windo,ws (2041–2060 and 2081–2100) under three SSP scenarios (SSP2-4.5, SSP3-7.0, SSP5-8.5) using the CRH TCGI. Cities span six basins: Eastern Pacific (EP), North Atlantic (NA), North Indian Ocean (NI), South Indian Ocean (SI), South Pacific (SP), and Western Pacific (WP). The central value is the multi-model median; values in parentheses show the range (minimum to maximum) across individual GCMs.

	basin	ERA5 [years]	base [years]	ssp245 [years]	2041–2060 ssp370 [years]	ssp585 [years]	ssp245 [years]	2081–2100 ssp370 [years]	ssp585 [years]
**Acapulco**	EP	4.7	5.5 (4.0–6.9)	5.5 (3.5–7.5)	8.5 (4.3–12.7)	7.7 (3.9–11.5)	7.9 (4.3–11.5)	8.9 (3.5–14.4)	8.9 (2.7–15.1)
**Manila**	WP	6.6	6.2 (5.2–7.1)	5.6 (5.1–6.0)	6.0 (4.4–7.6)	5.2 (4.8–5.6)	6.7 (5.3–8.1)	6.0 (5.3–6.8)	5.3 (3.9–6.7)
**Miami**	NA	8.7	8.7 (6.9–10.5)	8.3 (6.9–9.8)	7.4 (4.1–10.7)	7.8 (5.1–10.5)	9.7 (5.5–13.9)	10.7 (5.8–15.7)	10.4 (4.1–16.8)
**Mumbai**	NI	229.1	131.7 (88.5–174.8)	154.2 (84.1–224.3)	120.4 (82.8–157.9)	106.4 (59.1–153.8)	104.2 (64.7–143.7)	111.7 (70.2–153.2)	116.8 (43.2–190.5)
**Noumea**	SP	9.5	7.6 (6.3–8.8)	7.2 (5.8–8.5)	7.4 (6.2–8.6)	7.3 (6.2–8.4)	7.4 (5.7–9.1)	8.2 (5.9–10.5)	7.2 (4.9–9.4)
**Toamasina**	SI	7.8	6.4 (5.4–7.3)	5.8 (4.3–7.2)	368.8 (4.3–733.3)	6.3 (4.1–8.5)	7.1 (6.0–8.2)	655.2 (4.2–1306.1)	55.1 (3.9–106.3)

We conclude that our return period maps reproduce realistic spatial patterns in active basins and provide transparent uncertainty bounds for regions with infrequent storms, validating the dataset’s reliability for downstream impact and risk applications.

### Practical applications

Our technical validation demonstrates that the CHAZ-derived hazard maps can be directly used for various impact and risk analyses. The 100-year exceedance intensity and Category 1 return period examples illustrate how these layers apply to both present-day and future climate considerations. Importantly, in addition to the multi-model median fields shown in the maps, the tabulated minimum–maximum ranges (Tables 2, 3, Table S1, Table S2) provide a straightforward bound on intermodel spread, enabling users to bracket model uncertainty in their workflows. The full dataset also includes maps for additional return periods (10, 25, 50, 250, 1000 years) and higher thresholds (Category 3 winds), supporting a wide range of possible impact and risk applications.

Several parts of the hazard maps merit particular caution. First, we note that the gridded raster files (raster.nc) are generated by interpolating the point-based GeoDataFrames, produced by the local_exceedance_intensity and local_return_period methods, onto a regular 180-arcsecond spatial grid. However, this uniform grid may give a misleading impression of spatial precision over the ocean. In reality, the underlying centroids over ocean areas are spaced at a much coarser resolution of 3600 arcseconds (1 degree), leading to heavily interpolated values in those regions. As such, the dataset is best understood as a coastal hazard layer, and users are strongly advised against using values over the ocean, as the data there are too sparse to support meaningful applications. Additionally, in narrow coastal fringe cells where coarse ocean data are interpolated near finer land-based data, boundary artifacts may occur, further reducing confidence in those areas.

Second, poleward of the subtropics, the CHAZ event catalogue contains fewer storms, and those that do occur may already be undergoing extratropical transition; wind fields in these cells have therefore received less empirical calibration. Likewise, far-inland grid points experience relatively few synthetic storm passages, which limits their suitability for robust statistical analysis.

For example, while the CHAZ tracks themselves are well-resolved over the ocean, the gridded wind fields derived from them are limited by the coarser oceanic resolution. Consequently, the dataset is not suitable for offshore applications such as risk analysis for individual oil platforms, where small-scale features and localized winds are not resolved. By contrast, the dataset is well-suited for assessing hazard patterns and changes along coastlines, where event sampling is denser and spatial representation more robust. Further inland, however, values should be interpreted with caution: regions located hundreds of kilometers from the coast may still exhibit nonzero return periods or exceedance intensities, but these reflect very sparse event sampling and should not be taken at face value for quantitative risk assessments.

For most applications, we recommend that users compute multi-model averages over all six CMIP6 GCM-based runs. Individual-model files are recommended when inter-model differences themselves are of scientific interest. In all cases, we strongly advise keeping the two TCGI moisture variables (CRH and SD) separate. They represent alternative formulations of the genesis index that bracket plausible future activity and should be analyzed as bounding cases rather than averaged together, following the approach outlined by Lee *et al*.^15^.

## Supplementary information

Supplementary Information for article "Global coastal wind hazard maps from the CHAZ tropical cyclone model"

## Data Availability

The dataset is accessible via Columbia University’s Dryad repository: 10.5061/dryad.qfttdz0vz^31^ with the identifier 10.5061/dryad.qfttdz0vz, available under the Creative Commons Attribution 4.0 International (CC BY 4.0) license. The raw CHAZ TC track data used in this study are not currently hosted in a permanent repository. Researchers may contact the CHAZ development team at Columbia University for more information regarding data access (cl3225@columbia.edu).

## Code availability

For this study, we used the Python (version 3.11+) version of CLIMADA, release v6.0.2-dev^33^. Source code is openly and freely available under the terms of the GNU General Public License Version 3^4^. CHAZ is an open-source model, and its code can be downloaded from https://github.com/cl3225/CHAZ. Code to reproduce the results of this paper is available at a GitHub repository^34^ with the identifier 10.5281/zenodo.16058514.
