# Supplementary material for: Global coastal wind hazard maps from the CHAZ tropical cyclone model
Source: Sci Data. 2026 Jan 17;13:136. doi: 10.1038/s41597-025-06452-0 (PMC12858913; doi:10.1038/s41597-025-06452-0)
Supplement: Supplementary file 1 — Supplementary Information for article "Global coastal wind hazard maps from the CHAZ tropical cyclone model" [file 41597_2025_6452_MOESM1_ESM.pdf]

Supplementary Information for article "Global coastal wind hazard maps from the CHAZ tropical cyclone model"

Simona Meiler[1,2,*], Chia-Ying Lee[3], Suzana J. Camargo[3], and Adam H. Sobel[3,4]

[1]Civil and Environmental Engineering, Stanford University, USA
[2]Institute for Environmental Decisions, ETH Zurich, Switzerland
[3]Lamont-Doherty Earth Observatory, Columbia University, Palisades, NY, USA
[4]Department of Applied Physics and Applied Mathematics, Columbia University, New York, NY, USA
[*]Corresponding author: simona@simonameiler.ch

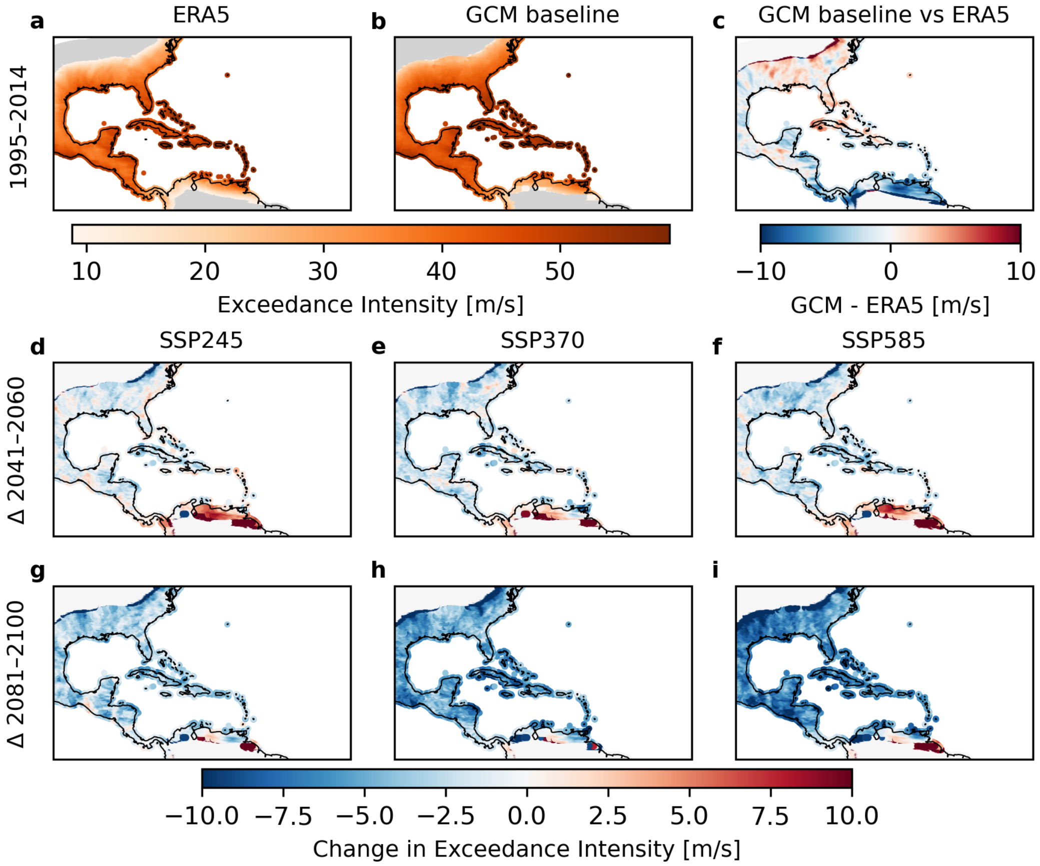

Fig. S1: 100-yr exceedance wind speeds (m/s) estimates for the Carribean - SD TCGI. The top row shows exceedance intensity estimates for the present-day baseline from ERA5 reanalysis (a), the GCM baseline (b), and difference between ERA5 and GCM baselines (c). The middle (bottom) row shows difference plots for the middle (end) of the century relative to the present-day baseline for three climate scenarios (SSP2-4.5 (d, g), SSP3-7.0 (e, h), SSP5-8.5 (f, i)). We computed the multi-model median from the six GCMs, using the SD TCGI.

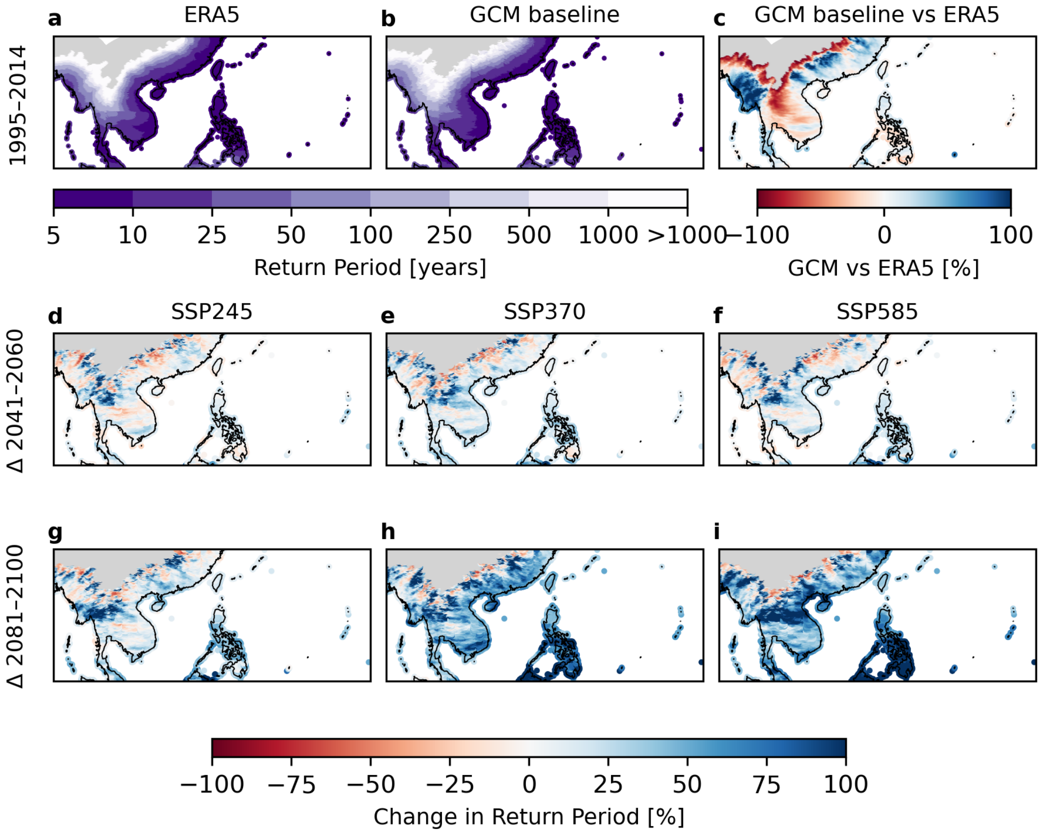

Fig. S2: Return period (years) estimates for Cat. 1 TC wind intensities in Southeast Asia - SD TCGI. The top row shows return period estimates for the present-day baseline from ERA5 reanalysis (a), the GCM baseline (b), and difference between ERA5 and GCM baselines (c). The middle (bottom) row shows difference plots for the middle (end) of the century relative to the present-day baseline for three climate scenarios (SSP2-4.5 (d, g), SSP3-7.0 (e, h), SSP5-8.5 (f, i)). We computed the multi-model median from the six GCMs, using the SD TCGI.

Table S1: 100-yr exceedance wind speeds (m/s) for six cities – SD TCGI. Listed are the modelled wind speeds corresponding to a 100-year return period in the historical period (ERA-5 and GCM baseline) and two future windows (2041–2060 and 2081–2100) under three SSP scenarios (SSP2-4.5, SSP3-7.0, SSP5-8.5) using the SD TCGI. Cities span six basins: Eastern Pacific (EP), North Atlantic (NA), North Indian Ocean (NI), South Indian Ocean (SI), South Pacific (SP), and Western Pacific (WP). The central value is the multi-model median; values in parentheses show the range (minimum to maximum) across individual GCMs.

	basin	ERA5 [m/s]	base [m/s]	2041–2060			2081–2100		
				ssp245 [m/s]	ssp370 [m/s]	ssp585 [m/s]	ssp245 [m/s]	ssp370 [m/s]	ssp585 [m/s]
**Acapulco**	EP	55.39	54.51 (51.05–56.02)	52.13 (46.24–56.44)	52.46 (45.74–59.18)	52.59 (48.82–56.36)	48.31 (40.97–55.66)	49.56 (39.14–58.10)	48.51 (35.32–53.59)
**Manila**	WP	51.59	52.23 (48.38–55.00)	53.98 (50.66–58.16)	53.70 (51.22–56.17)	52.95 (50.51–55.40)	52.05 (49.25–54.85)	49.68 (44.55–55.30)	49.78 (45.32–53.46)
**Miami**	NA	51.44	50.84 (48.50–53.99)	50.83 (46.89–53.07)	49.56 (46.49–52.63)	49.88 (46.12–53.65)	48.89 (44.50–53.28)	47.32 (42.98–52.53)	45.35 (40.00–49.69)
**Mumbai**	NI	27.83	32.01 (29.62–34.34)	32.02 (24.67–37.19)	29.42 (27.58–31.26)	30.31 (25.41–35.21)	30.64 (24.47–36.81)	27.07 (21.61–32.08)	25.86 (20.29–35.13)
**Noumea**	SP	52.96	54.08 (51.73–57.03)	52.91 (51.43–55.08)	52.37 (48.34–56.39)	52.26 (46.72–57.80)	53.84 (52.07–55.61)	50.71 (46.23–54.89)	50.16 (43.91–52.10)
**Toamasina**	SI	53.92	56.09 (54.24–58.01)	54.24 (52.81–57.06)	53.17 (49.99–56.36)	52.83 (50.17–55.48)	54.44 (52.28–57.04)	49.91 (44.07–50.98)	52.76 (44.93–54.04)

Table S2: Return period (years) estimates for Cat. 1 TC wind intensities for six cities – SD TCGI. Listed are the modelled return periods corresponding to a Cat. 1 TC wind intensity in the historical period (ERA-5 and GCM baseline) and two future windows (2041–2060 and 2081–2100) under three SSP scenarios (SSP2-4.5, SSP3-7.0, SSP5-8.5) using the SD TCGI. Cities span six basins: Eastern Pacific (EP), North Atlantic (NA), North Indian Ocean (NI), South Indian Ocean (SI), South Pacific (SP), and Western Pacific (WP). The central value is the multi-model median; values in parentheses show the range (minimum to maximum) across individual GCMs.

	basin	ERA5 [years]	base [years]	2041–2060			2081–2100		
				ssp245 [years]	ssp370 [years]	ssp585 [years]	ssp245 [years]	ssp370 [years]	ssp585 [years]
**Acapulco**	EP	4.7	6.8 (4.9–8.7)	8.3 (5.2–11.3)	10.6 (4.8–16.3)	8.5 (4.9–12.1)	13.4 (5.7–21.0)	19.6 (6.7–32.5)	34.3 (5.5–63.1)
**Manila**	WP	6.6	6.8 (5.2–8.3)	6.9 (5.7–8.1)	7.7 (5.8–9.6)	6.5 (5.4–7.7)	8.1 (5.9–10.2)	13.7 (8.2–19.1)	13.0 (6.7–19.3)
**Miami**	NA	8.7	9.0 (6.2–11.9)	9.1 (6.8–11.4)	11.7 (6.9–16.5)	9.4 (6.3–12.4)	15.9 (8.2–23.6)	15.9 (9.7–22.2)	22.3 (10.2–34.5)
**Mumbai**	NI	229.1	114.7 (78.7–150.7)	154.5 (77.9–231.2)	218.4 (121.8–314.9)	279.5 (72.3–486.7)	218.3 (69.3–367.4)	342.2 (115.2–569.1)	342.8 (86.3–599.3)
**Noumea**	SP	9.5	7.9 (6.3–9.4)	9.3 (6.6–11.9)	9.9 (7.0–12.8)	11.5 (6.6–16.4)	11.7 (8.2–15.2)	14.0 (9.5–18.5)	19.4 (9.1–29.8)
**Toamasina**	SI	7.8	5.9 (5.4–6.5)	7.8 (5.7–9.9)	7.8 (6.3–9.4)	9.5 (5.6–13.3)	10.4 (7.2–13.7)	19.8 (8.9–30.7)	18.1 (8.8–27.4)
